# Expression of *ANK3* moderates the association between childhood trauma and affective traits in severe mental disorders

**DOI:** 10.1038/s41598-023-40310-6

**Published:** 2023-08-24

**Authors:** Monica Aas, Ole A. Andreassen, Johannes Gjerstad, Linn Rødevand, Gabriela Hjell, Ingrid Torp Johansen, Synve Hoffart Lunding, Monica B. E. G. Ormerod, Trine V. Lagerverg, Nils Eiel Steen, Srdjan Djurovic, Ibrahim Akkouh

**Affiliations:** 1 Social, Genetic and Developmental Psychiatry Centre, Institute of Psychiatry, Psychology and Neuroscience, King’s College London, London, UK; 2 Department of Behavioural Sciences, OsloMet – Oslo Metropolitan University, Oslo, Norway; 3 NORMENT Centre for Psychosis Research, Oslo University Hospital and University of Oslo, Oslo, Norway; 4 Department of Psychiatry, Østfold Hospital, Grålum, Norway; 5 Department of Medical Genetics, Oslo University Hospital and University of Oslo, Oslo, Norway; 6 KG Jebsen Centre for Neurodevelopmental Disorders, University of Oslo, Oslo, Norway; 7 Department of Clinical Science, NORMENT, University of Bergen, Bergen, Norway

**Keywords:** Psychiatric disorders, Psychology

## Abstract

Exposure to early life trauma increases the risk of psychopathology later in life. Here we investigated if *ANK3* mRNA levels influence the relationship between childhood trauma experiences and clinical characteristics in mental disorders. A sample of 174 patients with bipolar disorder and 291 patients with schizophrenia spectrum disorder were included. Patients were diagnosed using the Structured Clinical Interview for DSM-IV, and childhood trauma was assessed using the childhood trauma questionnaire. Age at illness onset and number of psychotic and affective episodes were assessed from interview and medical records. Current depressive symptoms were measured using the calgary depression scale for schizophrenia and the inventory for depressive symptomatology. *ANK3* expression was analyzed in whole blood using the Illumina HumanHT-12 v4 Expression BeadChip. Analyses were carried out with the *Process* adjusted for confounders. Within the total sample, patients with both high *ANK3* expression and with the most severe childhood sexual abuse had more manic/hypomanic episodes and an earlier age at onset of the first episode. *ANK3* mRNA levels also moderated the relationship between emotional neglect and manic/hypomanic episodes. Our results suggest that *ANK3* expression levels moderate the association between specific types of childhood trauma and affective traits in mental disorders.

## Introduction

Childhood trauma is a well-known environmental stressor associated with mental illness, conferring a threefold increase in risk of both affective and psychotic disorders^1–4^. However, the biological mechanisms linking trauma to psychopathology are poorly understood. Increasing the knowledge of the long-term biological correlates of early life trauma in severe mental disorders is a pivotal step in the development of new and personalized treatment targets.

Studies indicate that childhood trauma may lead to long-lasting effects by interfering with developmental trajectories of various biological systems^5^. However, the factors determining why only some individuals develop psychopathology following childhood trauma experiences remain largely unknown. One plausible explanation is that the distinct trajectory from early adverse events to clinical end-points is largely shaped by epigenetic or other modifications regulating the expression of genes^6^. Given that individuals differ in their genetic make-up, they will also differ in their transcriptional response to environmental influences^6^. In addition to increasing the risk of affective and psychotic disorders, a history of childhood trauma is associated with an earlier age at onset and an increased number of episodes after illness onset in bipolar disorder (BD) and schizophrenia (SZ)^1,7,8^, also after adjusting for current mood at the time of the assessment^9^. Earlier onset is more often observed in patients with a severe illness trajectory, and these patients are more likely to have higher inherited risk than those with later onset^10^. Cumulative clinical episodes, especially within the first three years of the illness, are associated with increased risk of treatment resistance^11^.

It is therefore plausible that expression levels of genes associated with both psychiatric disease and exposure to early life trauma could be determinants of clinical outcomes.

One gene of relevance is *ANK3* (Ankyrin 3), which encodes a scaffolding protein localized to the axon and is required for the proper organization of ion channels^12^. Changes in *ANK3* expression have been persistently linked to experiences of early life adverse events as well as psychopathology, including BD^5^. Variants of *ANK3* have also consistently been identified as top hits in all three BD genome-wide association studies (GWAS) from the Psychiatric Genomics Consortium (PGC)^13–15^. Interestingly, *ANK3* may be associated with SZ and post-traumatic stress disorder (PTSD)^16,17^. Moreover, *ANK3* expression is associated with anhedonia and stress processing^18^, both of which are important features of traumatized patients including those with BD and SZ^8,19,20^.

BD and SZ are often viewed as part of the same continuum with shared neural, genetic and psychological mechanisms^21,22^, and both are associated with altered *ANK3* expression^16,18,23,24^. Animal and human studies have also demonstrated that early life stress may alter the methylation status of *ANK3*^5^, with behavioral correlates, further pointing to *ANK3* expression as a candidate moderator of early life stress effects in psychiatric diseases. A recent animal study also showed that repeated social defeat induces a persistent upregulation of *ANK3* mRNA in the pituitary gland^25^, strengthening a link between ANK3 expression and early adversities.

The main objective of the current study was to investigate how *ANK3* expression affects the association between childhood trauma and clinical manifestations such as age at onset and number of episodes of BD and SZ. Our overall hypothesis is that *ANK3* expression will moderate the association between childhood adverse events and clinical trait characteristics of BD and SZ, in particular affective symptoms, with and without adjusting for current depressive symptoms. Specifically, we hypothesize that patients with high *ANK3* mRNA levels and a history of childhood trauma experiences will have the earliest age at onset and the highest number of illness episodes.

## Methods

### Participants

Data were obtained from the ongoing Thematically Organized Psychosis (TOP) study at the Norwegian Center for Mental Disorders Research (NORMENT). Participants were enrolled between 2007 and 2021 from psychiatric units within the major hospitals in Oslo, Norway. For the current study, we included a total sample of n = 465 consisting of 291 patients with SZ spectrum disorder (schizophrenia, n = 164; schizophreniform, n = 24; schizoaffective, n = 46; other psychosis n = 57), and 174 patients with BD (bipolar 1, n = 135; bipolar II, n = 16; and not otherwise specified (NOS), n = 23), all recruited from the same catchment area. Exclusion criteria for patients were: age outside the range of 18–65 years, intellectual disability (IQ under 70), organic or substance-induced affective disorder or psychosis, or medical conditions interfering with brain function including neurological disorders, autoimmune diseases or cancer. Informed consent was obtained from all participants. The protocol for the study was approved by the South Eastern Norway Regional Committee for Medical Research Genetic Ethics (REK; 2009/2485/REK Sør-Øst). The Regional Committee for Medical Research Ethics and the Norwegian Data Inspectorate approved all aspects of the study. All methods were performed in accordance with the relevant guidelines and regulations.

### Clinical assessment

Participants were diagnosed by trained psychologists and medical doctors using the Structured Clinical Interview for DSM-IV Axis I disorders (SCID-I), chapters A-E^26^. Age at onset of first SCID-verified episode and number of episodes were assessed using SCID-I and medical records. Clinicians involved in the diagnostic ratings received regular clinical supervision from senior researchers and professors, both individually and in groups. A good inter-rater reliability for diagnostic assessments at the TOP study was indicated, with an overall kappa score between 0.92 and 0.99 across assessment teams^27^. Regular medication was presented as Daily Defined Dosage (DDD). In patients with a SZ spectrum diagnosis, we assessed depressive symptoms using the Calgary Depression Scale for Schizophrenia (CDSS)^28^, and in BD by the Inventory for Depressive Symptomatology (IDS)^29^. Information on alcohol use was obtain by the Alcohol use disorders identification test (AUDIT; http://whqlibdoc.who.int/hq/2001/who_msd_msb_01.6a.pdf. Information on smoking was defined as daily smoking yes, no. Childhood trauma was measured using the retrospective Childhood Trauma Questionnaire (CTQ)^30^. Childhood traumatic events were assessed as a continuous variable ranging from 25 to 125, with a higher score indicating greater severity^30^. Subtypes of sexual abuse, emotional abuse, physical abuse, emotional neglect and physical neglect were also investigated (scores ranging from a minimum score of 5 to a maximum score of 25 for each subtype). Moderate to severe cutoff scores from the CTQ manual are presented in Supplementary Material Table S1. A CTQ total score is defined as CTQ composite score throughout the study. Although other studies of childhood trauma have been published in overlapping datasets^7^, the association between *ANK3* expression and childhood trauma experiences within this study population has not yet been investigated.

### RNA microarray analysis and quality control

Blood sampling was done in the morning after one night fasting (median time of blood sampling was 9 AM). Quantification of mRNA levels was carried out using microarray-based gene expression analysis as described elsewhere^31^. Briefly, blood samples were collected in Tempus Blood RNA Tubes (Life Technologies Corporation). Total RNA was extracted with the TEMPUS 12-Port RNA Isolation Kit (Applied Biosystems) and ABI PRISM 6100 Nucleic Acid PrepStation (Applied Biosystems) according to manufacturer’s protocol. Gene expression analyses were performed with Illumina HumanHT-12 v4 Expression BeadChip (Illumina, Inc.). Multidimensional scaling and hierarchical clustering were used for regular quality control, including sample quality measurements and removal of outliers, as well as removal of multiple batch effects (RNA extraction batch, RNA extraction method, DNase treatment batch, cRNA labelling batch, and chip hybridization).

### Statistical analyses

Data were analyzed with IBM Statistics SPSS v27. Independent sample t-tests were performed for comparisons of demographic variables. The statistical modeling tool *Process v.3.5*^32^ was applied to investigate interaction effects between *ANK3* mRNA levels (moderator) and childhood trauma (independent variable) on clinical variables analyzed one at a time (as the dependent variable). The following clinical variables were investigated: current depressive symptoms, number of episodes (psychotic, elevated mood or depressive mood) and age at first episode. To control the Type I error rate we only investigated conditional effects if the interaction analysis was statistical significant. P-values presented were further adjusted for False Discovery Rate (FDR) and considered statistically significant if receiving a score of 1. All p-values in the result section are FDR-adjusted. Since a recent meta-analysis linked childhood abuse and childhood neglect to different symptoms in adults with a psychotic disorder^1^, the main analyses included different subtypes of trauma from the CTQ (physical abuse, sexual abuse, emotional abuse, physical neglect, and emotional neglect), as well as a CTQ total score. All variables in *Process* were entered as continuous variables. If the moderation analyses have a *p* value of < 0.05, *Process* calculates conditional effect of the predictor at the value of high, intermediate or low levels of the moderator, where high ANK3 group = 1 SD > mean levels, intermediate ANK3 group = mean ANK3 levels, and low ANK3 group = 1 SD < mean levels.

In the present study, interaction analyses and the conditional effects of the predictor at the different values of the moderator (high, intermediate, low) were run in *Process* with 5 000 bootstrap samples. As *Process* includes bootstrapping, it is a well-suited method for analyzing variables that are not normally distributed (including age at onset, and depressive symptoms from the CDSS and IDS). All statistical analyses involving *ANK3* expression measurements were based on the batch-adjusted log2-transformed data. Assumptions for regression analyses were checked and found satisfying. The main analyses were adjusted for age, sex, diagnosis, time of blood sampling, and medication use represented as defined daily dose (DDD) in accordance with the World Health Organization (WHO) guidelines (https://www.whocc.no/ddd/). We adjusted for duration of illness in the analyses of number of episodes. As current mood may affect retrospective information of childhood trauma events^33^, analyses were also conducted with and without adjustment for current depressive symptoms. As smoking status has been linked to changes in methylation and gene expression^34^, sensitivity analyses was also conducted adjusting for daily smoking (yes, no). A sample of 465 participants will have 80% power of detecting a medium effect, at a significance criterion of α = 0.05.

## Results

### Sample description

A total of 291 patients with SZ and 174 patients with BD were included (Table 1). The age at inclusion was 30.5 ± 10.2 (mean ± SD) years. The male-to-female ratio was 1.4 (56% males and 44% females). The sample consisted of 84.3% Europeans. The mean CTQ composite score was 43.84 ± 15.38 (range 25–117), with emotional neglect being the most frequently reported trauma reported by 29.3% of the patients. Adjusted for age, sex, and time of day of blood sampling, a trend was found for higher *ANK3* mRNA in BD compared to SZ (5.54 ± 0.38 and 5.47 ± 0.39, respectively, t = 1.90, *P* = 0.06). The mean age at onset in the total sample was 22.05 ± 9.04 and the average number of total episodes (including depressive, hypomanic/manic and psychotic episodes) were 5.47 ± 5.42. One hundred and fifty-four (74%) of BD patients had at least one psychotic episode. One hundred and thirty-six (47%) of the SZ spectrum group had at least one depressive episode, and 29 (10%) had at least one manic or hypomanic episode. No significant difference in current depressive symptoms or medication (DDD) was observed between the two patient groups, but patients with BD had more depressive and manic/hypomanic episodes compared to SZ. No association was observed between subtypes of medication (antipsychotic, mood, or antidepressants DDD) and ANK3 levels (*p* > 0.1, data not shown). Smoking status was associated with having lower ANK3 levels (current smokers, ANK3 mean ± SD = 5.46 ± 0.38 compared to 5.54 ± 0.40 in non-smokers, t = − 2.36, *p* = 0.009, CI =  = − 0.16–0.01).

**Table 1:** Clinical characteristics and sample overview.

	Total sample (n = 465)	SZ N = 291	BD N = 174	Statistics
Age, mean ± SD	30.48 ± 10.21	29.37 ± 9.13	32.33 ± 11.59	t = 3.06 *P* = 0.002
Female, N (%)	206 (44.30)	104 (35.70)	102 (58.60)	X^2^ = 23.11 *P* < .001
Europeans, N (%)	392 (84.30)	240 (82.50)	152 (87.40)	X^2^ = 1.96 *P* = .16
Marital status, married or live-in partner, N (%)	119 (25.60)	57 (19.60)	62 (35.60)	X^2^ = 14.72 *P* < *.001*
Employed or fulltime student, N (%)	149 (32.10)	80 (27.60)	69 (39.70)	X^2^ = 361.03 *P* < *.001*
Daily tobacco users, N (%)	277 (59.57)	179 (62.40)	98 (56.30)	X^2^ = 1.65 *P* = .20
Audit score, mean ± SD	7.31 ± 6.71	7.04 ± 6.93	7.78 ± 6.31	t = 73 *P* = .14
Education, years, mean ± SD	12.56 ± 2.64	12.17 ± 2.72	13.22 ± 2.36	t = .57 *P* = .28
CTQ, mean ± SD	43.84 ± 15.38	44.25 ± 15.09	43.19 ± 15.83	t = .68 *P* = .50
Physical abuse, mean ± SD	6.90 ± 3.50	6.93 ± 3.58	6.84 ± 3.36	t = .27 *P* = .79
Sexual abuse, mean ± SD	6.60 ± 3.52	6.46 ± 3.22	6.84 ± 3.96	t = .38 *P* = .70
Emotional abuse, mean ± SD	10.56 ± 5.18	10.63 ± 10.44	10.44 ± 5.43	t = .90 *P* = .37
Physical neglect, mean ± SD	7.81 ± 3.02	7.93 ± 2.94	7.61 ± 3.15	t = 1.10 *P* = .27
Emotional neglect, mean ± SD	11.92 ± 5.01	12.08 ± 4.96	11.64 ± 5.10	t = -.68 *P* = .50
ANK3*, mean ± SD	5.49 ± 0.39	5.47 ± 0.39	5.54 ± 0.38	t = 1.90 *P* = .06
DDD, mean ± SD	1.66 ± 1.81	1.69 ± 2.05	1.61 ± 1.29	t = .49 *P* = .63
Depressive symptoms#, mean ± SD	.00 ± 1.00	.05 ± 1.00	− .05 ± 1.01	t = 1.08 *P* = .28
AAO, mean ± SD	22.05 ± 9.04	21.54 ± 8.39	22.85 ± 9.94	t = 1.64 *P* = .12
AAO Depression, mean ± SD	21.33 ± 8.56	20.70 ± 7.62	21.98 ± 9.39	t = 1.29 *P* = .20
AAO Mania/hypomania, mean ± SD	24.79 ± 9.57	22.34 ± 5.78	25.70 ± 10.13	t = 2.25 *P* = .03
AAO psychosis	24.44 ± 8.84	23.55 ± 8.20	26.37 ± 9.86	t = 3.21 *P* = .0.03
Total episodes, mean ± SD	5.47 ± 5.42	3.48 ± 3.79	8.35 ± 6.10	t = 9.63 *P* < .001
Depression episodes, mean ± SD	2.85 ± 4.33	1.73 ± 3.25	4.64 ± 5.17	t = 6.51 *P* < .001
Mania/hypomania episodes, mean ± SD	1.77 ± 4.03	.42 ± 1.99	4.01 ± 5.36	t = 8.22 *P* < .001
Psychosis episodes, mean ± SD	1.58 ± 1.96	1.60 ± 1.59	1.53 ± 2.40	t = .39 *P* = *.72*

*SZ* schizophrenia spectrum, *BD* bipolar disorder, *CTQ* childhood trauma questionnaire, *DDD* daily defined dose; * ANK3 mRNA adjusted for age, sex and time of blood sample. #Depressive symptoms were assessed by the z scores of Calgary Depression Scale for Schizophrenia (CDSS) and The Inventory for Depressive Symptomatology (IDS) for bipolar disorder. *AAO* age at onset, *SD* standard deviation, *Audit* alcohol use disorders identification test.

Patients with BD were more likely to be married and working or fulltime student compared to patients with SZ, while no difference was observed for daily tobacco use or alcohol use from the Audit.

Patients with higher *ANK3* mRNA also showed a trend of more manic/hypomanic episodes and earlier age at onset of mania/hypomania (see Supplementary Material Tables S2–S3).

### *ANK3* expression, childhood trauma and clinical characteristics

**Figure 1:**
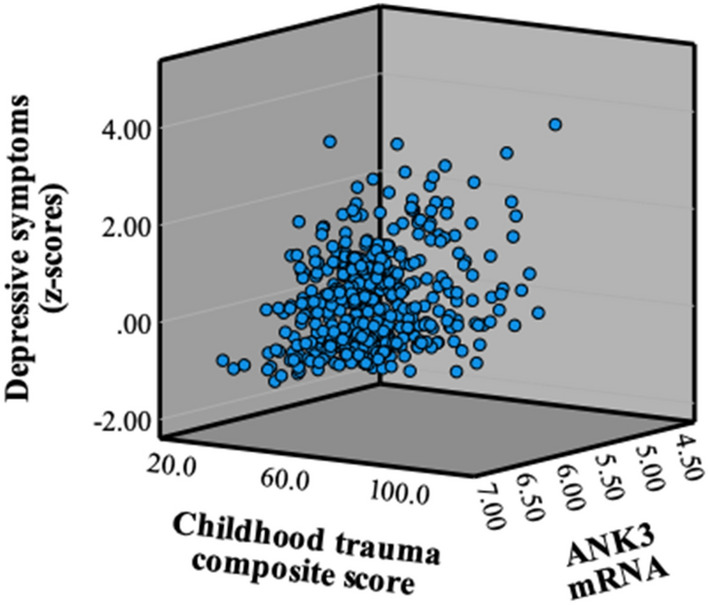
*ANK3* mRNA, childhood trauma composite score and depressive symptoms. *Process*, moderation analysis. *ANK3* mRNA levels moderated the association between CTQ composite score and current depressive symptoms (ß_CTQ composite score × ANK3_ = .01, *p* < .05. Depressive symptoms were assessed by the z-scores of Calgary Depression Scale for Schizophrenia (CDSS) and The Inventory for Depressive Symptomatology (IDS) for bipolar disorder.

Within the total sample, *ANK3* mRNA levels moderated the association between CTQ composite score and current depressive symptoms (ß_sexual abuse × *ANK3*_0.01, *p* < 0.05, CI =  < 0.01–0.003; Fig. 1 r^2^ = 0.01).

**Figure 2:**
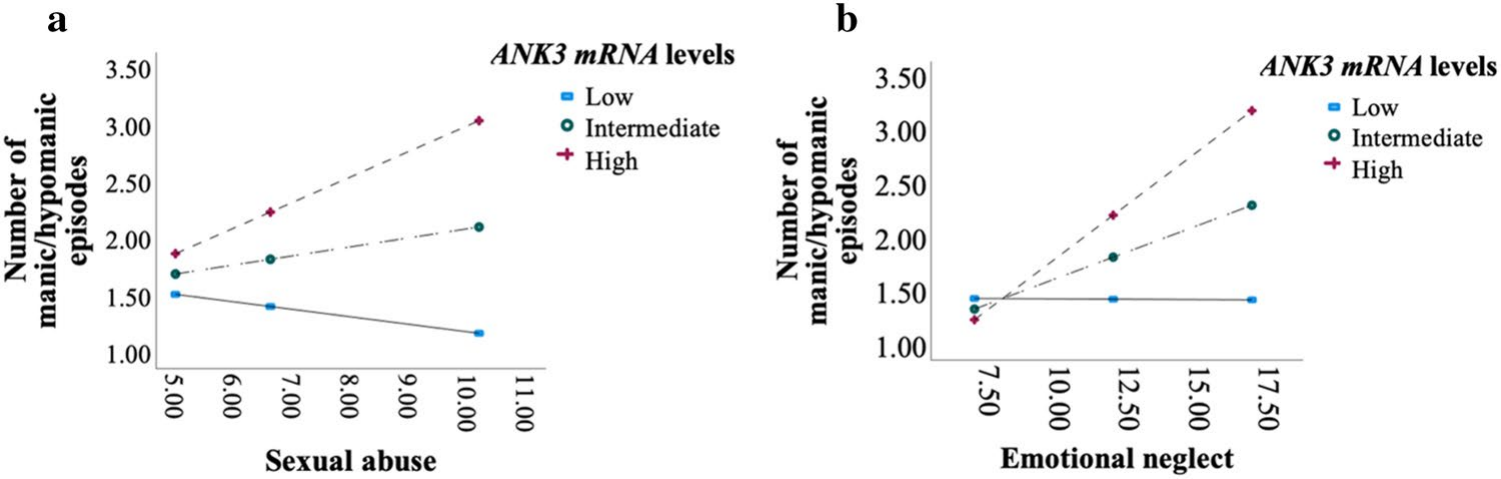
(**a**–**b**) *ANK3* mRNA moderates the relationship between sexual abuse, emotional neglect, and elevated mood episodes. *Process*, moderation analysis. Sexual abuse: ß = .43, *p* = .003. Emotional neglect: ß = .24, *p* = .0009. Adjusted for age, sex, medication, Daily Defined Dose, diagnosis (schizophrenia spectrum/bipolar disorders), duration of illness, and time of blood sample. High *ANK3* group = 1 SD > mean levels. Intermediate *ANK3* group = mean *ANK3* levels, and Low *ANK3* group = 1 SD < mean levels.

*ANK3* mRNA moderated the effects of childhood sexual abuse and emotional neglect on number of manic/hypomanic episodes (ß_sexual abuse × *ANK3*_ = 0.43, *P* = 0.003, CI = 0.14–0.71; r^2^ = 0.01 and ß_sexual abuse × *ANK3*_ = 0.24, *P* = 0.0009, CI = 0.06–0.042; r^2^ = 0.01 respectively; Table 2). Patients with high *ANK3* mRNA and with the most severe levels of sexual abuse or emotional neglect had more manic/ hypomanic episodes (ß = 0.27; *p* = 0.0005, CI = 0.12–0.42; and ß = 0.21; *p* < 0.001 CI = 0.11–0.31; respectively, Fig. 2a, b). No significant associations were observed for depressive or psychotic episodes (p > *0.1)*.

**Table 2:** ANK3 mRNA moderates the association between subtypes of trauma and clinical traits.

	Number of manic or hypomanic episodes					Age at onset manic or hypomanic episode				
	ß	t	se	*P*	*CI*	ß	t	se	*P*	*CI*
CTQ total										
Interaction	.04	1.28	.03	.20	− .02 to − .10	− .04	− .48	.09	.63	− .21 to − .13
Physical abuse										
Interaction	− .06	− .56	.11	.58	− .27 to − .15	− .12	− .29	.41	.77	− .92 to − .68
Sexual abuse										
Interaction	.43	2.97	.15	.003	.14 to − .71	− 1.23	− 2.59	.46	.006	− .2.10 to − .36
Conditional effect										
High ANK3	.27	3.50	.08	.0005	.12 to − .42	− .64	− 2.56	.25	.01	− 1.14 to − .15
Intermediate ANK3	.10	1.97	.05	.05	.001 to − .20	− .16	− .91	.17	.36	− .49 to − .18
Low ANK3	− .07	− .90	.08	.36	− .22 to − .08	.33	1.40	.24	.16	− .13 to − .80
Emotional abuse										
Interaction	.09	1.06	.09	.29	− .08 to − .27	.11	.44	.26	.66	.39 to − .62
Physical neglect										
Interaction	.04	.23	.18	.81	− .30 to − .38	.27	.53	.50	.60	− .73 to 1.26
Emotional neglect										
Interaction	.24	2.52	.09	.0009	.06 to − .42	− .20	− .76	.26	.45	− .70 to − .31
Conditional effect										
High ANK3	.21	4.21	.05	< 0.001	.11 to − .31					
Intermediate ANK3	.12	3.38	.04	.0008	.05 to − .19					
Low ANK3	.03	.52	.05	.60	− .07 to − .12					

*AAO* age at onset, *CTQ* childhood trauma questionnaire. Moderation analyses performed using *Process* with bootstrapping. Analysis adjusted for the time of the blood sample, diagnosis, sex, age and medication (total defined daily dose). Trauma data are all continuous. mRNA data are continuous. High ANK3 group = 1 SD > mean levels. Intermediate ANK3 group = mean ANK3 levels and Low ANK3 group = 1 SD < mean levels.

To control the Type I error rate we only investigated conditional effects if the interaction analysis was statistical significant.

**Figure 3:**
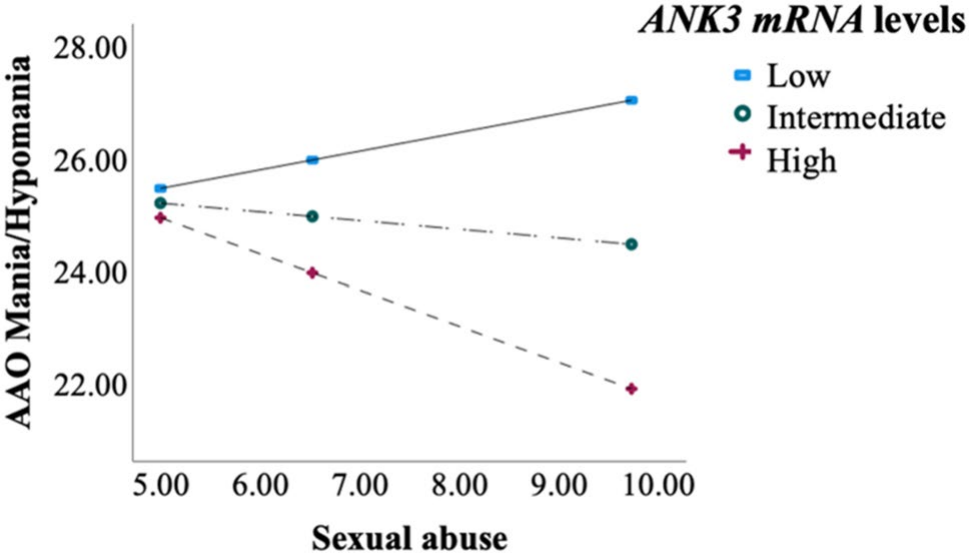
*ANK3* mRNA moderates the relationship between sexual abuse and earlier age at onset of mania/hypomania. *Process*, moderation analysis. ß = − 1.23, *p* = .006. Adjusted for age, sex, medication, Daily Defined Dose, diagnosis (schizophrenia spectrum/bipolar disorders) and time of blood sample. High *ANK3* group = 1 SD > mean levels. Intermediate *ANK3* group = mean *ANK3* levels, and Low *ANK3* group = 1 SD < mean levels, *AAO* = age at onset.

*ANK3* mRNA levels moderated the association between childhood sexual abuse and age at onset of mania/hypomania (ß_sexual abuse × *ANK3*_ = − 1.23, *p* = 0.006, CI = − 2.10 to − 0.36; r^2^ = 0.02). Patients with high *ANK3* mRNA levels and severe scores of sexual traumas had the earliest age at onset of mania/hypomania (ß = − 0.64; *P* = 0.01, CI = − 1.14 to − 0.15; see Fig. 3). No statistically significant relationship between *ANK3* expression, childhood traumas and age at first psychotic or depressive episode (*P* > 0.05).

### Clinical traits adjusting for current depressive symptoms

Sensitivity analyses adjusting for current depressive symptoms showed that *ANK3* expression moderated the relationship between specific types of childhood traumatic events (sexual abuse and neglect) and number of mania/hypomanic episodes and corresponding age at onset (see Table 3) also after taking into consideration current depressive symptoms. Findings above remained unchanged when smoking status was added into the model (data not shown).

**Table 3:** ANK3 mRNA moderates the association between subtypes of trauma and clinical traits adjusted for current depressive symptoms.

	Number of manic or hypomanic episodes					Age at onset manic or hypomanic episode				
	ß	t	se	*P*	CI	ß	t	se	*P*	CI
CTQ total										
Interaction	.03	.95	.03	.34	− .03 to − .09	.01	.09	.09	.93	− .17 to − .19
Physical abuse										
Interaction	− .07	− .67	.11	.51	− .28 to − .14	− 0.01	− 0.3	.41	.98	− 8.95 to − 4.05
Sexual abuse										
Interaction	.39	2.71	.14	.007	.11 to − .67	− 1.28	− 2.86	0.45	.005	− 2.16 to − .40
Conditional effect										
High ANK3	.25	3.22	.08	.001	.10 to − .41	− .61	− 2.39	.26	.02	− 1.12 to − .11
Intermediate ANK3	.08	1.58	.05	.12	− .02 to − .19	− .08	− .46	.17	.65	− .42 to − .26
Low ANK3	− .04	− .53	.07	.60	− .18 to − .10	.40	1.70	.24	.09	− .06 to − .87
Emotional abuse										
Interaction	.08	.88	.09	.38	− .10 to − .25	.24	.90	.27	.37	− .29 to − .78
Physical neglect										
Interaction	− .04	− .24	.17	.81	− .39 to − .30	.59	1.13	.52	.26	− .43 to − 1.61
Emotional neglect										
Interaction	.20	2.16	.09	.03	.02 to − .38	− .06	− .21	.27	.83	− .59 to − .47
Conditional effect										
High ANK3	.18	3.45	.05	.006	.08 to − .29					
Intermediate ANK3	.09	3.53	.04	.12	.02 to − .17					
Low ANK3	.03	.67	.05	.54	− .07 to − .13					

*Process.* *CTQ* childhood trauma questionnaire. Moderation analyses performed using *Process* with bootstrapping. Adjusted for the time of the blood sample, diagnosis, sex, age and medication (total defined daily dose) and current depressive symptoms. Depressive symptoms were assessed by the z scores of Calgary Depression Scale for Schizophrenia (CDSS) and The Inventory for Depressive Symptomatology (IDS) for bipolar disorder. Trauma data are all continuous. mRNA data are continuous. Data not shown: No significant findings for age at first psychotic or first depressive episode, or number of psychotic or depressive episodes (n.s.).

To control the Type I error rate we only investigated conditional effects if the interaction analysis was statistical significant.

## Discussion

We found that *ANK3* mRNA levels moderated the relationship between specific types of childhood traumas and affective episodes in severe mental disorders. Within the total sample, Patients with both high *ANK3* expression and with the most severe childhood sexual abuse and emotional neglect had more manic/hypomanic episodes and an earlier age at onset of the first episode. No relationship was observed between number of psychotic or depressive episodes and *ANK3* mRNA levels.

A recent meta-analysis of childhood trauma in psychosis empathized that biological mechanisms linking trauma to clinical presentations are lacking (Alameda et al.^1^). Our study suggests an interplay between *ANK3* mRNA levels and childhood trauma and affective episodes. Affective symptoms are the most consistent clinical dimension associated with childhood trauma experiences^1^. As previously discussed, childhood trauma is a well-known environmental stressor associated with mental illness, conferring a threefold increase in risk of both affective and psychotic disorders^1–4^. As previously reported in partly overlapping samples^7,35^, our current study showed that patients with childhood trauma experiences had more severe clinical features, including more mood episodes and an earlier age at onset of affective episodes (see Supplementary Material Tables S1, S2). However, the factors determining why only some individuals develop psychopathology following childhood trauma experiences remain largely unknown. One plausible explanation is that the expression of genes that shape the trajectory from early adverse events to clinical end-points^6^. Large GWAS studies in severe mental disorders demonstrate an altered *ANK3* expression in this group compared to healthy individuals^14,15^, and our study suggests an interplay between *ANK3* mRNA levels and childhood trauma exposure which increases vulnerability to develop affective symptoms, as is also supported by previous studies showing a direct link between early stress and ANK3 levels^5,25^.

Exposure to stress or early adversities both after and in the perinatal period has been linked to changes in the methylation status of the *ANK3* gene^5^, which is supported by RNA changes in animals exposed to stress^25^. Furthermore, in humans an interaction has been observed between a polymorphism affecting *ANK3* and obstetric complications impacting connectivity during a working memory task often found to be impaired in mental disorders^5^. As discussed by Luoni and colleagues^5^, ANK3 may be an important mediator between early life stress and psychopathology by changing neuronal circuits important for psychiatric illness. Since ANK3 expression can be modified by pharmacological interventions and by long-term lithium use, ANK3 could be seen as a target for drug intervention in individuals with stress-related disorders with neurodevelopmental origin, but more research is needed in this field.

While our study has several strengths, such as a well characterized clinical sample with detailed information on gene expression and clinical characteristics, it also has some limitations. First, history of childhood trauma was measured retrospectively by asking adults about experiences in their childhood. A recent meta-analysis showed low overlap between prospective and retrospective data of childhood trauma^33^, and current mood at the time of assessment may contribute to over or under-reporting of retrospective experiences^36^. As discussed by^33^ retrospective data may have higher sensitivity in detecting actual cases that otherwise could go unnoticed, but reliance solely on retrospective assessment methods might also lead to misclassification of exposed groups^33,37,38^. Therefore, improving the reliability of retrospective data, such including sensitivity analyses adjusting for current depressive symptoms level, is warranted which we have done in this study. Second, *ANK3* expression was measured in whole blood, which may not wholly recapitulate expression patterns in brain. However, previous studies have demonstrated a significant up-regulation of *ANK3* in peripheral blood from BD patients^23,39^, which is consistent with the predicted direction of effect of the protective *ANK3* variant associated with BD^23^. This suggests that peripheral expression of *ANK3* may be an acceptable proxy for brain expression. The role of physical health as well as information from healthy controls should be further addressed in future studies. Our study specifically focused on ANK3 mRNA expression as the gene has been found to be associated with both BD and schizophrenia in large GWAS^14,16^, and studies have also shown that ANK3 is influenced by early trauma experiences^5,18^. Future studies should also investigate in larger samples a broader specter of gene expression and its alteration by childhood trauma in severe mental disorders. In summary, our study points to *ANK3* mRNA expression as a biological marker influencing the relationship between childhood trauma experiences and more severe affective clinical traits in severe mental disorders.

### Supplementary Information

Supplementary Information.

## Data Availability

Data available on request (for meta-analysis etc.) from the corresponding author. Data were analyzed using Process, see link: https://www.processmacro.org/index.html.

## References

[1] Alameda L, Christy A, Rodriguez V, Salazar de Pablo G, Thrush M, Shen Y (2021). Association between specific childhood adversities and symptom dimensions in people with psychosis: Systematic review and meta-analysis. Schizophr. Bull..

[2] Alameda L, Levier A, Gholam-Rezaee M, Golay P, Vandenberghe F, Delacretaz A, Baumann P (2020). Psychological trauma occurring during adolescence is associated with an increased risk of greater waist circumference in Early Psychosis patients treated with psychotropic medication. PLoS ONE.

[3] Etain B, Aas M (2021). Childhood maltreatment in bipolar disorders. Curr. Top. Behav. Neurosci..

[4] Varese F, Smeets F, Drukker M, Lieverse R, Lataster T, Viechtbauer W (2012). Childhood adversities increase the risk of psychosis: A meta-analysis of patient-control, prospective- and cross-sectional cohort studies. Schizophr. Bull..

[5] Luoni A, Massart R, Nieratschker V, Nemoda Z, Blasi G, Gilles M (2016). Ankyrin-3 as a molecular marker of early-life stress and vulnerability to psychiatric disorders. Transl. Psychiatry.

[6] Nöthling J, Malan-Müller S, Abrahams N, Hemmings SMJ, Seedat S (2020). Epigenetic alterations associated with childhood trauma and adult mental health outcomes: A systematic review. World J. Biol. Psychiatry.

[7] Aas M, Dieset I, Morch R, Steen NE, Hope S, Reponen EJ (2019). Reduced brain-derived neurotrophic factor is associated with childhood trauma experiences and number of depressive episodes in severe mental disorders. Schizophr. Res..

[8] Aas M, Pizzagalli DA, Laskemoen JF, Reponen EJ, Ueland T, Melle I (2019). Elevated hair cortisol is associated with childhood maltreatment and cognitive impairment in schizophrenia and in bipolar disorders. Schizophr. Res..

[9] Aas M, Ueland T, Lagerberg TV, Melle I, Aminoff SR, Hoegh MC (2022). Retrospectively assessed childhood trauma experiences are associated with illness severity in mental disorders adjusted for symptom state. Psychiatry Res..

[10] Hilker R, Helenius D, Fagerlund B, Skytthe A, Christensen K, Werge TM, Nordentoft M, Glenthøj B (2017). Is an early age at illness onset in schizophrenia associated with increased genetic susceptibility? Analysis of data from the nationwide Danish twin register. EBioMedicine.

[11] Berk M, Post R, Ratheesh A, Gliddon E, Singh A, Vieta E (2017). Staging in bipolar disorder: From theoretical framework to clinical utility. World Psychiatry.

[12] Ferreira MA, O’Donovan MC, Meng YA, Jones IR, Ruderfer DM, Jones L (2008). Collaborative genome-wide association analysis supports a role for ANK3 and CACNA1C in bipolar disorder. Nat. Genet..

[13] Sklar P, Ripke S, Scott LJ, Andreassen OA, Cichon S, Psychiatric GWAS Consortium Bipolar Disorder Working Group (2011). Large-scale genome-wide association analysis of bipolar disorder identifies a new susceptibility locus near ODZ4. Nat. Genet..

[14] Stahl EA, Breen G, Forstner AJ, McQuillin A, Ripke S, Trubetskoy V (2019). Genomewide association study identifies 30 loci associated with bipolar disorder. Nat. Genet..

[15] Mullins, N. *et al.* Genome-wide association study of more than 40,000 bipolar disorder cases provides new insights into the underlying biology. *Nat. Genet.* **53**, 817–829 (2021). 10.1038/s41588-021-00857-4 PMC8192451 34002096

[16] Hughes T, Hansson L, Sønderby IE, Athanasiu L, Zuber V, Tesli M (2016). A Loss-of-function variant in a minor isoform of ANK3 protects against bipolar disorder and schizophrenia. Biol. Psychiatry.

[17] Logue MW, Solovieff N, Leussis MP, Wolf EJ, Melista E, Baldwin C, Koenen KC, Petryshen TL, Miller MW (2013). The ankyrin-3 gene is associated with posttraumatic stress disorder and externalizing comorbidity. Psychoneuroendocrinology.

[18] Leussis MP, Berry-Scott EM, Saito M, Jhuang H, de Haan G, Alkan O (2013). The ANK3 bipolar disorder gene regulates psychiatric-related behaviors that are modulated by lithium and stress. Biol. Psychiatry.

[19] Horan WP, Kring AM, Blanchard JJ (2006). Anhedonia in schizophrenia: A review of assessment strategies. Schizophr. Bull..

[20] Rizvi SJ, Lambert C, Kennedy S (2018). Presentation and neurobiology of anhedonia in mood disorders: Commonalities and distinctions. Curr. Psychiatry Rep..

[21] Smeland OB, Bahrami S, Frei O, Shadrin A, O'Connell K, Savage J (2020). Genome-wide analysis reveals extensive genetic overlap between schizophrenia, bipolar disorder, and intelligence. Mol. Psychiatry.

[22] Sorella, S. *et al.* Testing the expanded continuum hypothesis of schizophrenia and bipolar disorder. Neural and psychological evidence for shared and distinct mechanisms. *Neuroimage Clin.* **23**, 101854 (2019). 10.1016/j.nicl.2019.101854 PMC6529770 31121524

[23] Hughes T, Sønderby IE, Polushina T, Hansson L, Holmgren A, Athanasiu L (2018). Elevated expression of a minor isoform of ANK3 is a risk factor for bipolar disorder. Transl. Psychiatry.

[24] Roussos P, Katsel P, Davis KL, Bitsios P, Giakoumaki SG, Jogia J, Rozsnyai K, Collier D, Frangou S, Siever LJ, Haroutunian V (2012). Molecular and genetic evidence for abnormalities in the nodes of ranvier in schizophrenia. Arch. Gen. Psychiatry.

[25] Olsen MB (2022). Mapping of pituitary stress-induced gene regulation connects Nrcamto negative emotions. iScience.

[26] Spitzer, R. L., Williams, J. B., Gibbon, M. & First, M. B. The Structured Clinical Interview for DSM-III-R (SCID). I: History, rationale, and description. *Arch. Gen. Psychiatry* **49**, 624–629 (1992). 10.1001/archpsyc.1992.01820080032005 1637252

[27] Høegh MC, Melle I, Aminoff SR, Laskemoen JF, Büchmann CB, Ueland T, Lagerberg TV (2020). Affective lability across psychosis spectrum disorders. Eur. Psychiatry..

[28] Addington D, Addington J, Schissel B (1990). A depression rating scale for schizophrenics. Schizophr. Res..

[29] Rush AJ, Giles DE, Schlesser MA, Fulton CL, Weissenburger J, Burns C (1986). The inventory for depressive symptomatology (IDS): Preliminary findings. Psychiatry Res..

[30] Bernstein DP, Fink L, Handelsman L, Foote J, Lovejoy M, Wenzel K (1994). Initial reliability and validity of a new retrospective measure of child abuse and neglect. Am. J. Psychiatry.

[31] Akkouh IA, Ueland T, Andreassen OA, Brattbakk HR, Steen VM, Hughes T (2018). Expression of TCN1 in blood is negatively associated with verbal declarative memory performance. Sci. Rep..

[32] Hayes, A. F. PROCESS: A versatile computational tool for observed variable mediation, moderation, and conditional process modeling [White paper]. Retrieved from http://www.afhayes.com/public/process2012.pdf (2012).

[33] Baldwin JR, Reuben A, Newbury JB, Danese A (2019). Agreement between prospective and retrospective measures of childhood maltreatment: A systematic review and meta-analysis. JAMA Psychiat..

[34] Kopa PN, Pawliczak R (2018). Effect of smoking on gene expression profile—overall mechanism, impact on respiratory system function, and reference to electronic cigarettes. Toxicol. Mech. Methods.

[35] Etain B, Aas M, Andreassen OA, Lorentzen S, Dieset I, Gard S (2013). Childhood trauma is associated with severe clinical characteristics of bipolar disorders. J. Clin. Psychiatry.

[36] Dalgleish, T. & Werner-Seidler, A. Disruptions in autobiographical memory processing in depression and the emergence of memory therapeutics. *Trends. Cogn. Sci.* **18**, 596–604 (2014). 10.1016/j.tics.2014.06.010 25060510

[37] Newbury JB, Arseneault L, Moffitt TE, Caspi A, Danese A, Baldwin JR, Fisher HL (2018). Measuring childhood maltreatment to predict early-adult psychopathology: Comparison of prospective informant-reports and retrospective self-reports. J. Psychiatr. Res..

[38] Reuben A, Moffitt TE, Caspi A, Belsky DW, Harrington H, Schroeder F, Hogan S, Ramrakha S, Poulton R, Danese A (2016). Lest we forget: Comparing retrospective and prospective assessments of adverse childhood experiences in the prediction of adult health. J. Child Psychol. Psychiatry.

[39] Wirgenes KV, Tesli M, Inderhaug E, Athanasiu L, Agartz I, Melle I, Hughes T, Andreassen OA, Djurovic S (2014). ANK3 gene expression in bipolar disorder and schizophrenia. Br. J. Psychiatry.

