# Supplementary material for: Expression of ANK3 moderates the association between childhood trauma and affective traits in severe mental disorders
Source: Sci Rep. 2023 Aug 24;13:13845. doi: 10.1038/s41598-023-40310-6 (PMC10449847; doi:10.1038/s41598-023-40310-6)
Supplement: Supplementary file 1 — Supplementary Information. [file 41598_2023_40310_MOESM1_ESM.docx]

**Supplementary Material**

**Figure S1.** Diagram Moderation analysis in *Process*


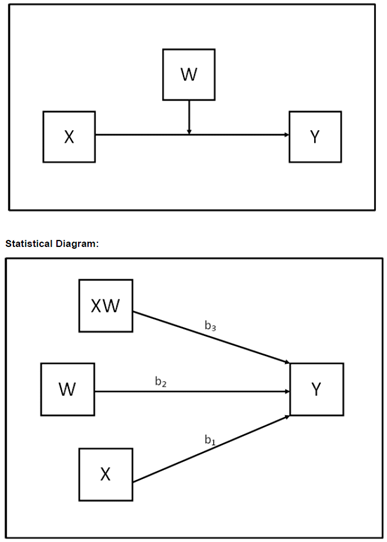


The Predictor (X) is childhood trauma cumulative or subtypes; ANK3 is the moderator (W) and the outcome (Y) is the clinical characteristics (number of episodes, age at onset etc).

**Table S1.** Childhood trauma subtypes and moderate to severe cutoff score

| **Physical abuse, N (%)** | **70 (15.2)** |
| --- | --- |
| **Sexual abuse, N (%)** | **86 (19.1)** |
| **Emotional abuse, N(%)** | **125 (27.8)** |
| **Physical neglect, N (%)** | **114 (25.0)** |
| **Emotional neglect, N (%)** | **132 (29.3)** |

**Table S2.** Childhood trauma, *ANK3* mRNA and current depressive symptoms and number of episodes

|  | *Depressive symptoms from IDS or CDSS | Number of manic/ hypomanic episodes | Number of depressive episodes | Number of psychotic episodes |
| --- | --- | --- | --- | --- |
|  | ß, t, se, p, CI | ß, t, se, p, CI | ß, t, se, p, CI | ß, t, se, p, CI |
| **CTQ total** | .31, 7.01, .003, <.001, .15-.027 | .14, 2.76, .01 .006, .1-.07 | .18, 3.60, .003, <.001, .026-.08 | .01, 1.85, .10, .07, -.001-.03 |
| **Physical abuse** | .21, 4.80, .01, <.001, .035-.09 | .04, .74, .06, .46, -.07-.15 | .06, 1.28, .01, .20, -.04-.20 | .02, .39, .03, .70, -.04-.06 |
| **Sexual abuse** | .20, 4.46, .01, <.001, .03-.09 | .12, 2.57, .06, .01, .03-.25 | .08, 1.64, .06, .10, -.02-.23 | .09, 1.83, .03, .07, -.004-.10 |
| **Emotional abuse** | .35, 8.21, .01, <.001, .05-.09 | .15, 3.15, .04, .002, .05-.20 | .24, 5.10, .01, <.001, .12-.28 | .03, .59 .02, .56, -.03-.05 |
| **Physical neglect** | .19, 4.41, .02, <.001, .04-.11 | .06, 1.22, .07, .22, -.05-.21 | .11, 2.33, .07, .02, .03-.31 | .11, 2.24, .03, .03, -.01-.13 |
| **Emotional neglect** | .23, 5.36, .01, <.001, .03-.07 | .11, 2.33, .04, .02. .02-.17 | .13, 2.77, .04, .006, .03-.20 | .10, 1.98, .02, <.05, <.01-.07 |
| **ANK3 mRNA** | -.03, -.71, .11, .48, -.34-.13 | .09, 2.11, .43, .04, .06-1.77 | -.02, -.39, .49, .70. -1.56-.78 | .05, 1.04, .23, .30,-.22-.70 |

Linear regression analysis. CTQ= Childhood Trauma Questionnaire. *Depressive symptoms were assessed by the z-scores of Calgary Depression Scale for Schizophrenia (CDSS) and The Inventory for Depressive Symptomatology (IDS) for bipolar disorder. *ANK3* mRNA analyses adjusted for the time of the blood sample, diagnosis, sex, age and medication (total defined daily dose).

**Table S3.** Childhood trauma, *ANK3* mRNA and age at onset

|  | AAO depressive episode | AAO manic or hypomanic episode | AAO psychosis |
| --- | --- | --- | --- |
|  | ß, t, se, p, CI | ß, t, se, p, CI | ß, t, se, p, CI |
| **CTQ total** | -.18, -3.05, .001, .003, -.01- -.002 | -.13, -1.74, .002, .08, -.01-<.01 | -.05, -1.02, .03, .31, -.004-.001 |
| **Physical abuse** | -.04, -0.64, .01, .52, -.02-.01 | .01, 0.11, 0.01, .91, -.02-.02 | .-03, -.57, .12, .57, -.01-.01 |
| **Sexual abuse** | -.13, -2.20, .01, .03, -.02--.001 | -.09, -1.19, .01, .24, -.03-.01 | -.06, -1.10, .13, .27, -.02-.003 |
| **Emotional abuse** | -.25, -4.31, .004, <.001,-.02--.01 | -.14, -1.91, .01, .06, -.02-<.01 | -.06, -1.15, .09, .25, -.01-.003 |
| **Physical neglect** | -.10, -1.78, .01, .08, -.02-.001 | -.07, -.91, .01, .36, -.03-.01 | -.01, -.20, .15, .85, -.01-.01 |
| **Emotional neglect** | -.15, -2.47, .004, .01, -.02- -.002 | -.12, -1.63, .01, .11, -.02-.002 | -.03, -.51, .09, .61, -.01-.01 |
| **ANK3 mRNA** | -.05, -1.26, .10, .21, -.07-.10 | -.11, -1.92, 1.37, .06, -.25- .02 | .002, .05, .73, .96, -.04 -.09 |

Linear regression analysis. CTQ= Childhood Trauma Questionnaire. *ANK3* mRNA analyses adjusted for the time of the blood sample, diagnosis, sex, age and medication (total defined daily dose). AAO=age at onset.
